# Nomogram for prediction of plastic bronchitis in Chinese children with pneumonia

**DOI:** 10.3389/fped.2025.1571479

**Published:** 2025-05-13

**Authors:** Xiaoqian Fang, Hemin Lu

**Affiliations:** Department of Pediatrics Department, Dongyang People’s Hospital, Dongyang, Zhejiang, China

**Keywords:** pneumonia, plastic bronchitis, risk factors, nomogram, atelectasis, mycoplasma

## Abstract

**Background:**

This study investigated risk factors for plastic bronchitis (PB) in children with pneumonia and created a nomogram for early detection.

**Methods:**

We retrospectively analyzed data from 487 children with pneumonia who underwent bronchoscopic alveolar lavage between 2018 and 2024. Children were divided into a PB group (*n* = 65) and a No-PB group (*n* = 422). Key indicators were identified using regression analysis, and a nomogram prediction model was developed. Its effectiveness was evaluated using receiver operating characteristic (ROC) curves, calibration curves, decision curve analysis (DCA), and the bootstrap (BS) method.

**Results:**

A total of 65 patients (13.3%) out of 487 had PB. Multivariate logistic regression analysis indicated that mycoplasma infection, atelectasis, high fever peak, high neutrophil levels and elevated lactate dehydrogenase levels were risk factors for PB development. Additionally, the BS-ROC curve of the developed nomogram had an area under the curve value of 0.857 (95% confidence interval: 0.808–0.905). The calibration curve analysis revealed a strong agreement between the actual and predicted probabilities of PB development, and DCA highlighted the clinical relevance of the nomogram.

**Conclusions:**

A nomogram for MP infection, fever peak, LDH, maximum neutrophils percentage (N%max), and atelectasis was established to predict the risk of PB in children with pneumonia. The nomogram effectively identifies PB early, and bronchoscopy is advised for high-risk children. However, the nomogram needs external validation before practical use.

## 1 Introduction

Approximately 344 million patients with lower respiratory tract infections are reported each year, resulting in approximately 502,000 fatalities among children under fiver years of age, making it the second highest cause of death in this demographic (1, 2). Airway mucus hypersecretion is a pathological change associated with pneumonia. If it develops as a bronchial mucus thrombus or leads to plastic bronchitis (PB), it can cause clinical relapse, delayed radiographic resolution, and long-term pulmonary sequelae (3, 4). Therefore, searching for specific clinical features and related markers in children with pneumonia is crucial to determine whether they develop PB at an early stage. Currently, models are related to PB caused by a single pathogen, such as mycoplasma (MP) infection (3, 4). However, increasing evidence also reports models on PB caused by other pathogens, including adenovirus (ADV), respiratory syncytial virus (RSV), bocavirus, and *Haemophilus influenzae* (5–9). Clinically, children with mixed infections were more prevalent than single infections, occurring in 46.7% of cases vs. 40.7% (10). Consequently, we did not classify the children based on a specific pathogen; instead, we included all children with pneumonia. A risk model for PB in children with pneumonia was constructed by identifying relevant markers based on clinical features and laboratory tests.

## 2 Methods

### 2.1 Participants

**Figure 1:**
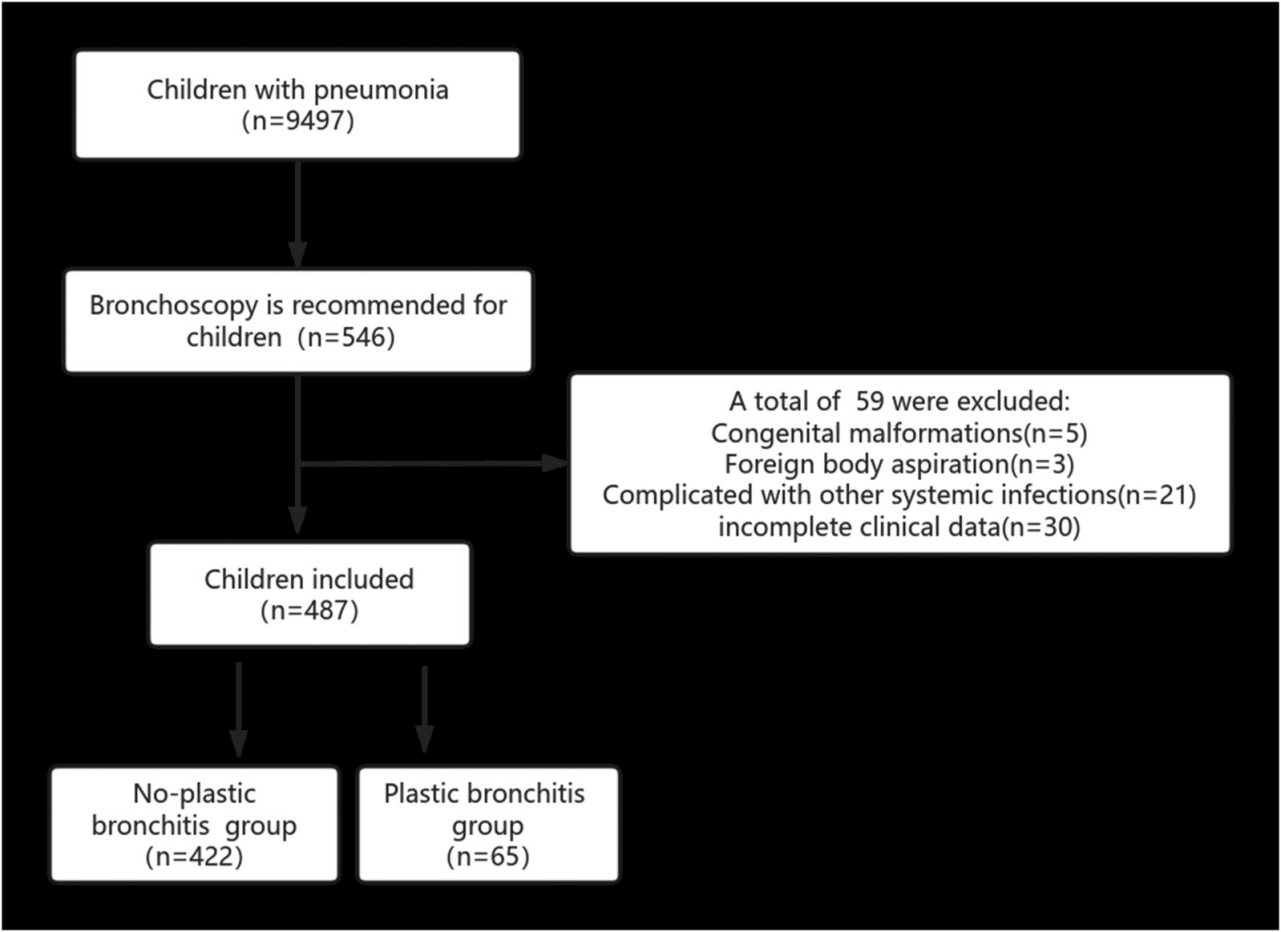
Flowchart of this study.

Patients who met the inclusion criteria at the Dongyang City People's Hospital from April 2018 to August 2024 were retrospectively reviewed. The inclusion criteria for patients were as follows: (1) Hospitalized in our hospital and were ≤14 years old; (2) diagnosed with pneumonia and treated with bronchoscopic alveolar lavage; (3) whose family members agreed and signed an informed consent. The exclusion criteria for patients were as follows: (1) Congenital malformations, including respiratory, circulatory, and urinary malformations; (2) congenital genetic diseases or chromosomal abnormalities; (3) complicated by infections in other parts, such as enteritis and infectious mononucleosis; (4) foreign body aspiration; (5) incomplete clinical data. Figure 1 presents a flowchart of our research.

This retrospective study was approved by the Ethics Committee of Dongyang People's Hospital (Approval No: Dong Ren Yi 2024-YX-156) and required written informed consent from the parents for each case.

### 2.2 Definition of plastic bronchitis

PB diagnosis was based on BAL condition. PB is characterized by the production of sticky, branching casts within the tracheobronchial tree, which can cause partial or complete airway obstruction. These casts can range from small, segmental casts within the bronchus to large ones that fill the entire airway (11).

### 2.3 Data collection

Two physicians gathered clinical data, blood tests, and alveolar lavage fluid results from all eligible children. These data included information on gender, age, body mass index (BMI), duration and peak of fever, pulmonary signs, whether corticosteroid was used before bronchoscopy, blood routine examination, coagulation function, pharyngeal pathogenic bacterial polymerase chain reaction (MP, ADV, RSV), alveolar lavage fluid culture, bronchoalveolar lavage fluid (BAL) MP, and PB presence, among others. Pleural effusion and atelectasis were diagnosed by radiologists and pediatricians based on lung CT findings.

### 2.4 Statistical analysis

Statistical analysis was performed using R (version 4.4.2) software. Continuous data are expressed as mean ± standard deviation or median and interquartile range, with a *t*-test or rank sum test applied depending on the data distribution. Categorical data are expressed as numbers (%) and analyzed using the chi-square or Fisher's exact test. The least absolute shrinkage and selection operator (LASSO) regression was used to screen for the most variable indices. Multivariate logistic regression was used to identify the primary risk factors, and a two-way method was used to determine independent risk factors. Based on these analyses, a nomogram was constructed. Receiver operating characteristic curves, calibration curves, and decision curve analysis (DCA) were calculated using bootstrapping (BS) (1,000 resamplings) to assess the predictive power and performance of the model. A *P* < 0.05 was considered statistically significant.

## 3 Results

### 3.1 Clinical characteristics of patients with PB

A total of 487 children were included in our study, of whom 65 developed PB and were included in the positive group (PB group), while the remaining 422 were included in the negative group (No-PB group). Statistically significant differences were observed between the two groups in fever duration, peak fever, atelectasis, pleural effusion, MP infection (pharynx and BAL), maximum neutrophils percentage (N%max), lymphocytes (L%max), eosinophilic granulocytes (E%max), platelet minimum, alanine aminotransferase, aspartate aminotransferase, and lactate dehydrogenase (LDH). However, no statistical differences were observed in other factors, such as corticosteroid use before bronchoscopy, age, BMI, and gender (Table 1).

**Table 1:** Differential analysis of two groups.

Variables	No-PB group	PB group	*p* Value
	(*n* = 422)	(*n* = 65)	
Gender			0.381
Female	236 (55.92%)	32 (49.23%)	
Male	186 (44.08%)	33 (50.77%)	
BMI	15.34 (14.31;17.16)	15.20 (14.24;16.46)	0.654
Age (month)	84.26 (33.17)	88.03 (25.13)	0.285
Duration of fever(day)	6.00 (4.00;7.00)	7.00 (6.00;8.00)	0.001*
Peak fever (°C)	39.50 (39.00;39.90)	40.00 (39.50;40.20)	<0.001*
Corticosteroid before bronchoscopy			0.660
NO	337 (79.86%)	54 (83.08%)	
YES	85 (20.14%)	11 (16.92%)	
Rale			1.000
NO	322 (76.30%)	50 (76.92%)	
YES	100 (23.70%)	15 (23.08%)	
Atelectasis			<0.001*
NO	413 (97.87%)	54 (83.08%)	
YES	9 (2.13%)	11 (16.92%)	
Pleural effusion			<0.001*
NO	360 (85.31%)	42 (64.62%)	
YES	62 (14.69%)	23 (35.38%)	
MP.BAL			0.004*
NO	113 (26.78%)	6 (9.23%)	
YES	309 (73.22%)	59 (90.77%)	
MP			<0.001*
NO	96 (22.75%)	2 (3.08%)	
YES	326 (77.25%)	63 (96.92%)	
Bacterial infection			0.213
NO	339 (80.33%)	57 (87.69%)	
YES	83 (19.67%)	8 (12.31%)	
ADV			1.000
NO	408 (96.68%)	63 (96.92%)	
YES	14 (3.32%)	2 (3.08%)	
RSV			0.437
NO	419 (99.29%)	64 (98.46%)	
YES	3 (0.71%)	1 (1.54%)	
WBCmax	9.55 (7.38;12.62)	10.57 (8.47;13.93)	0.033*
Lymphocyte(%).max	34.60 (28.20;43.88)	29.20 (24.70;33.40)	<0.001*
Monocyte(%).max	9.10 (7.60;11.00)	9.00 (7.00;11.60)	0.902
Neutrophil(%).max	68.90 (61.82;76.60)	75.70 (67.90;82.10)	<0.001*
Eosinophilic granulocyte(%).max	1.70 (0.70;3.20)	0.90 (0.40;2.90)	0.111
Platelet. min	251.00 (206.00;319.00)	220.00 (189.00;274.00)	0.004*
Hemoglobin.min	118.01 (8.84)	118.74 (8.97)	0.546
CRP.max	18.06 (8.04;37.01)	24.60 (12.81;46.89)	0.006*
INR	1.02 (0.98;1.07)	1.04 (0.99;1.08)	0.117
PT	13.30 (12.90;13.90)	13.50 (13.10;13.90)	0.234
APTT	38.30 (35.42;41.30)	37.30 (34.40;40.00)	0.075
TT	16.10 (15.60;16.80)	16.10 (15.60;16.70)	0.297
CK-MB	24.00 (20.00;30.00)	24.00 (18.00;35.00)	0.971
ALT	13.00 (10.00;17.00)	15.00 (12.00;18.00)	0.010*
AST	28.00 (24.00;34.00)	31.00 (26.00;39.00)	0.002*
LDH	312.00 (265.00;361.75)	380.00 (313.00;468.00)	<0.001*
BUN	3.69 (3.01;4.41)	3.79 (3.04;4.42)	0.809

Data are presented as median [IQR], Mean (SD), *n* (%).

.max, maximum;.min, minimum; PB, plastic bronchitis; BMI, body mass index; MP, mycoplasma; BAL, bronchoalveolar lavage fluid; ADV, adenovirus; RSV, respiratory syncytial virus; WBC, white blood cell; CRP, C-reactive rrotein; INR, international normalized ratio; PT, prothrombin time; APTT, activated partial thromboplastin time; TT, thrombin time; CK-MB, creatine kinase-MB; ALT, alanine aminotransferase; AST, aspartate aminotransferase; LDH, lactate dehydrogenase; BUN, blood urea nitrogen.

* Statistically significant difference.

### 3.2 LASSO regression analysis of PB

**Figure 2:**
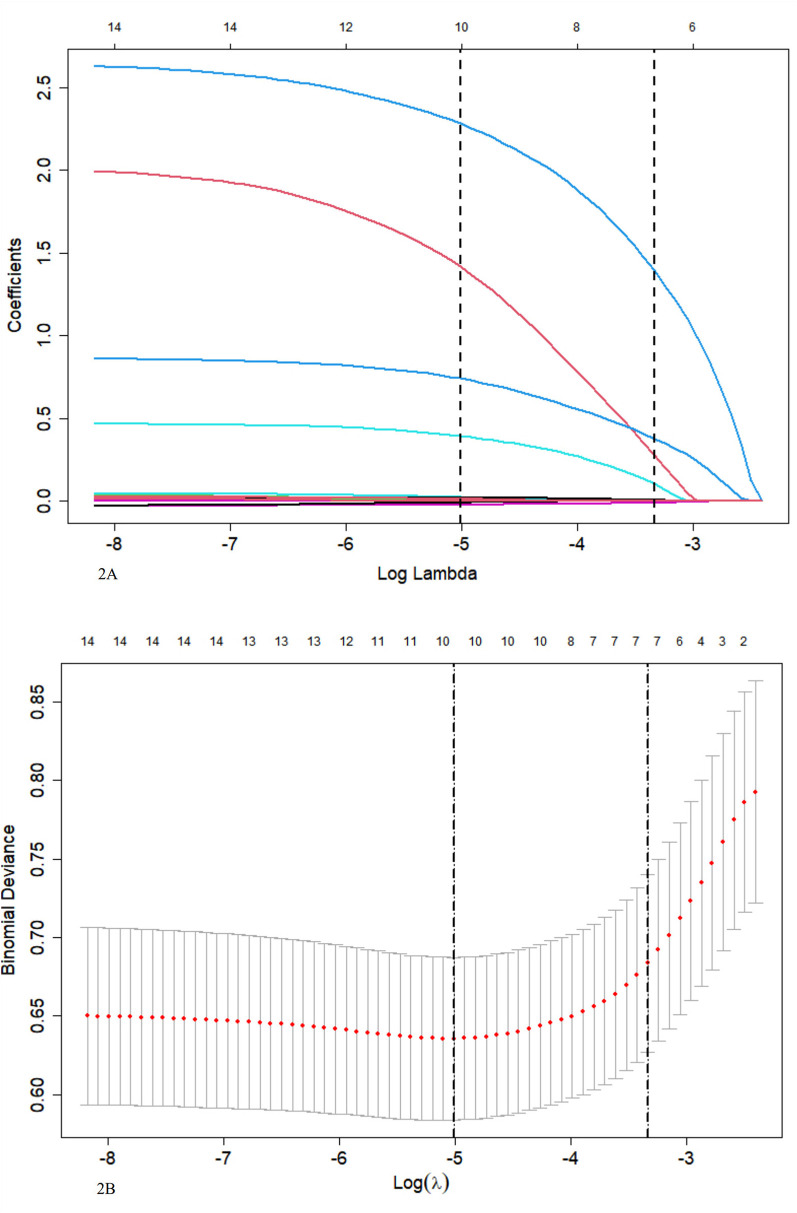
**(A)** LASSO coefficient profile for 14 variables indicates that more coefficients shrink to zero as penalties increase. **(B)** Tenfold cross-validation and minimization criteria were used to select the optimal penalty, lambda. The binomial deviance curve was plotted against the log of lambda, with vertical lines indicating 1 standard error. Seven variables with non-zero coefficients were selected with the optimal lambda.

Seven indexes were identified by LASSO regression analysis: Peak temperature, atelectasis, pleural effusion, L%max, N%max, MP infection, and LDH (Figures 2A,B). Due to the clear correlation between L%max and N%max, we've opted to include N%max, as it is widely used in clinical practice.

### 3.3 Logistic regression analysis of PB

**Figure 3:**
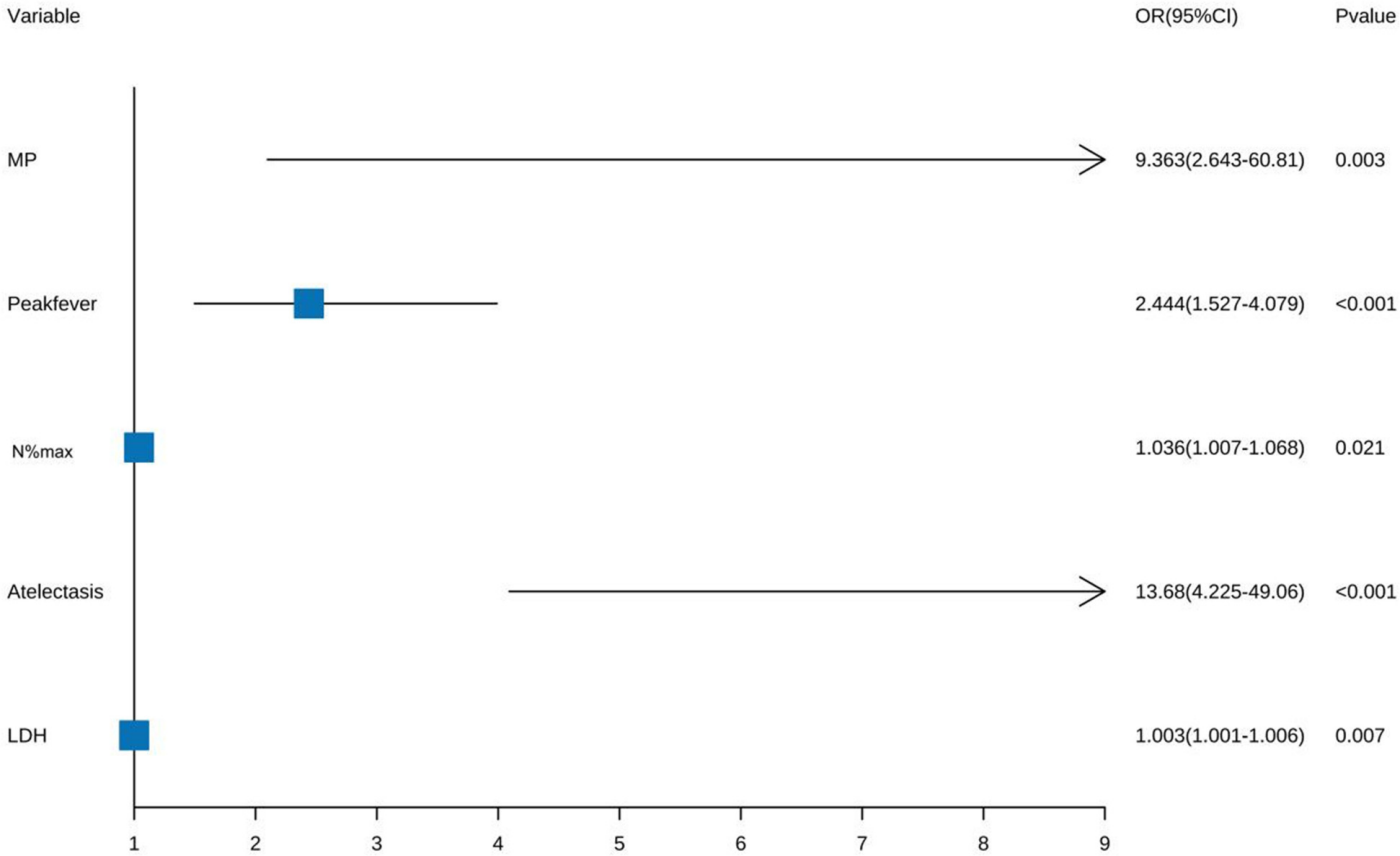
Logistic regression analysis conducted on PB risk factors.

Five factors were identified by multivariate logistic regression: Peak fever [odds ratio (OR) = 2.444, 95% confidence interval (CI): 1.527–4.079; *P* < 0.001], atelectasis (OR = 13.68, 95% CI: 4.225–49.06; *P* < 0.001), MP infection (OR = 9.363, 95% CI: 2.643–60.81; *P* = 0.003), N%max (OR = 1.036, 95% CI: 1.007–1.068; *P* = 0.021), and LDH (OR = 1.003, 95% CI: 1.001–1.006; *P* = 0.007) were independent risk factors. Pleural effusion was excluded (Figure 3).

### 3.4 Creation and verification of a predictive model for PB

**Figure 4:**
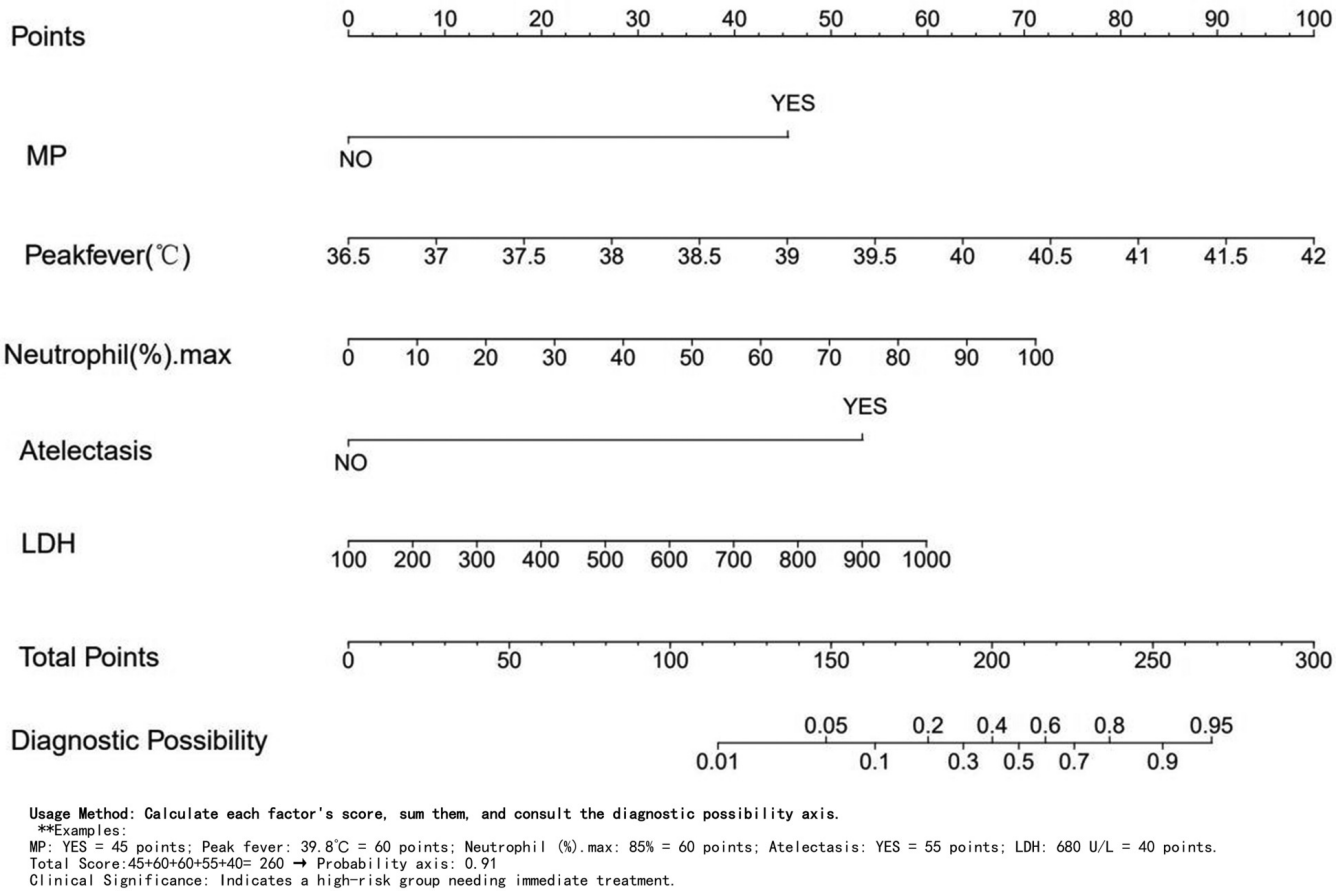
Nomogram for predicting PB.

**Figure 5:**
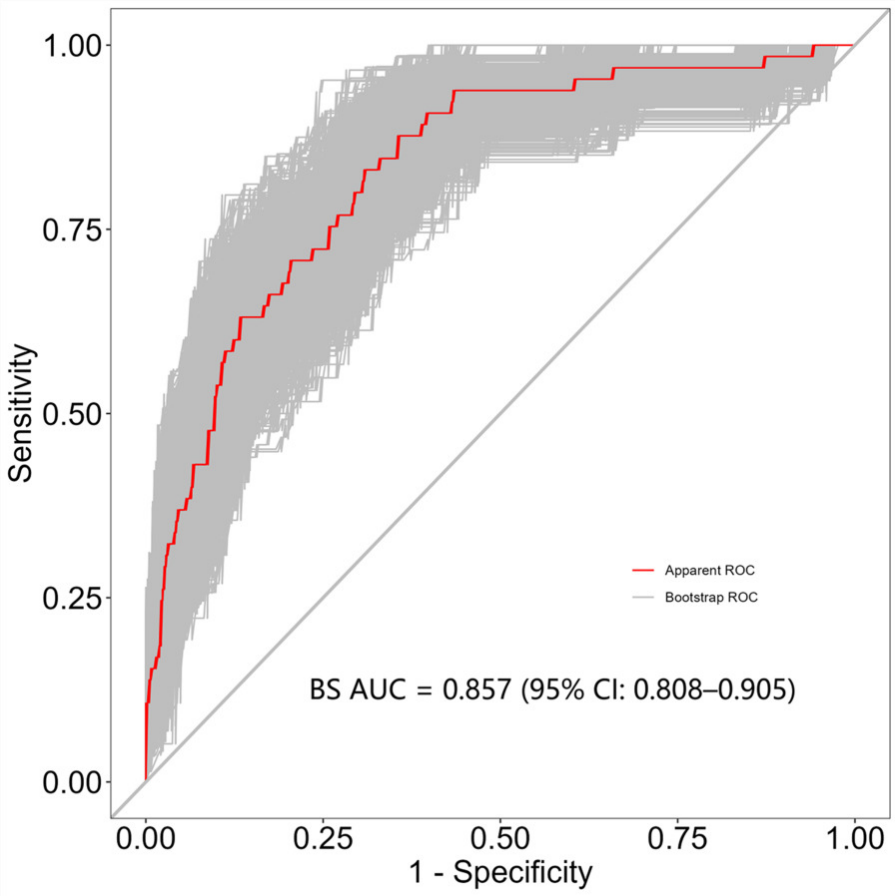
BS-ROC curve of the prediction model.

**Figure 6:**
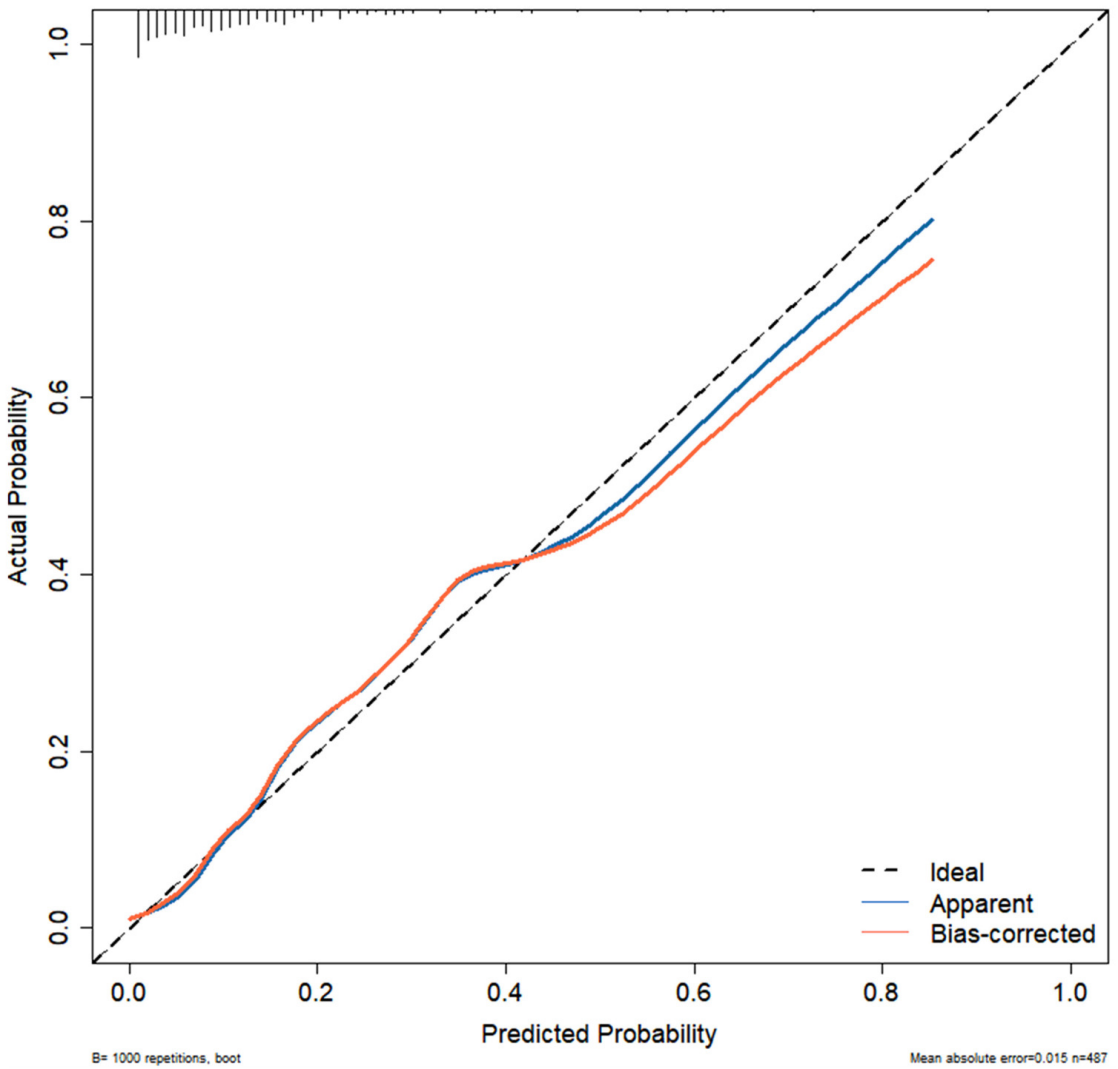
Calibration curves of nomogram.

**Figure 7:**
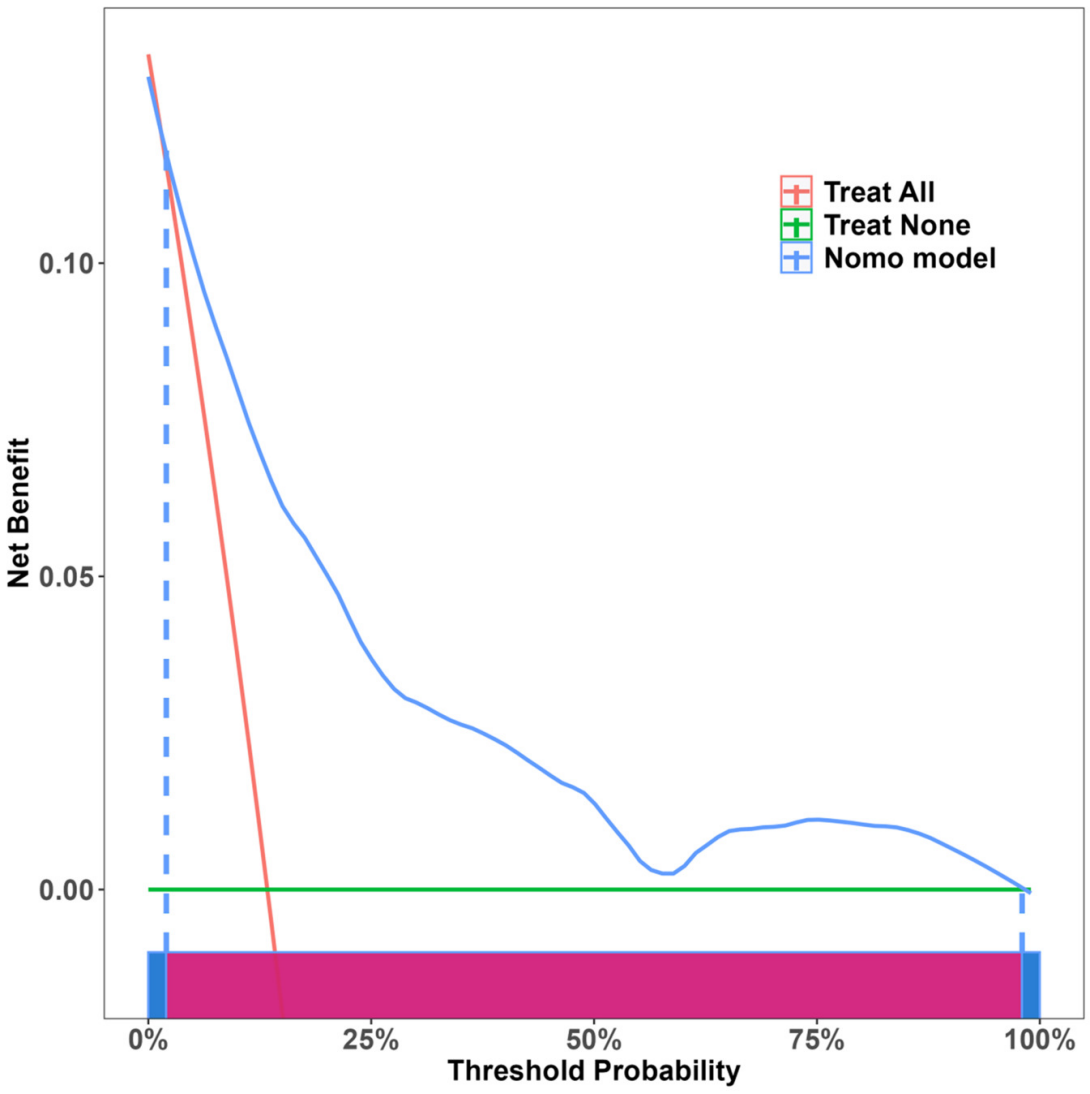
DCA of nomogram.

Independent risk factors from multivariate logistic regression analysis were used to create a nomogram (Figure 4). Each predictor score in the nomogram was summed to obtain a total score, which was then used to determine the likelihood of PB occurrence, aiding in evaluating the clinical necessity of aggressive bronchoscopic intervention. Our analysis demonstrated substantial predictive accuracy and model discrimination, with BS area under the curve values of 0.857 (95% CI: 0.808–0.905) obtained from 1,000 resamplings (Figure 5). Furthermore, the calibration curve of the nomogram demonstrated a reasonably a good match between the actual occurrence of PB and the predicted likelihood, with a *P*-value of 0.21 from the Hosmer–Lemeshow test confirming this finding (Figure 6). The DCA of the nomogram indicated that net benefits were achieved over a threshold probability range of 0.02–0.98. These findings suggest that the model may be an excellent predictor of PB-complicated pneumonia in children (Figure 7).

## 4 Discussion

Infection is a major element in PB formation (11, 12). During the acute phase of childhood pneumonia, persistent mucus accumulation can lead to PB, resulting in partial or complete dysfunction of pulmonary ventilation, dyspnea, recurrent high fever, pleural effusion in some cases, atelectasis, and even respiratory failure requiring mechanical ventilation, which can be dangerous to life in severe conditions or result in long-term effects (5, 12, 13). Therefore, early recognition of PB is significant. However, most existing models focus on single pathogens, although clinical cases often present with mixed infections. Consequently, we included all children with pneumonia and searched for relevant specific markers based on clinical characteristics and laboratory tests to construct a risk model for PB in children with pneumonia.

The MP infection independently influenced the PB development in our model, unlike ADV, RSV, or bacterial infections.This process is likely to cause PB for the following reasons: (1) MP infection directly adheres to airway epithelial cells and causes damage via cytotoxic effects or immune mechanisms. (2) MP-induced PB development in mouse models is associated with selective modulation of lymphatic structural changes through the PI3K/AKT/mTOR signaling cascade and pathways involving VEGFR-3 (14). Bronchial mucus plug or PB models based on MP infection in children have been previously reported in the literature (3, 15, 16), whereas MP infection remains one of the primary pathogens associated with PB in mixed infections. The literature indicates that 13 out of 15 cases (86.7%) with bronchoscopic treatment for childhood complex PB were MP-positive (17). In addition, MP infection and other pathogenic bacteria can cause PB. Huang (12) reported 43 cases of PB, of which 14 were MP-positive and 19 were ADV-positive. Chen et al. (18) reported that the top three pathogens in 55 cases of PB were MP (41.8%), ADV (20.0%), and influenza B virus (10.9%). However, our model did not identify ADV infection as a risk factor for PB. This process may be related to the restricted number of ADV infection cases in our group, which comprised only 14 cases, or to the number of ADV vectors. It has been reported that only one-third of children with ADV-infected pneumonia have bronchial mucus embolism, and the ADV number in the alveolar lavage fluid of these children is significantly increased. In mixed infections, particularly those involving ADV combined with MP, the incidence of bronchial mucus embolism is significantly higher (5). The influenza virus is a common cause of PB (18), but we excluded it from our model due to a lack of routine testing. There are limited clinical reports on PB resulting from bacterial or RSV infections, and no models exist for PB caused by a single pathogen, highlighting the need for more research.

Our research indicates that high neutrophil levels increase the risk of developing PB, whereas high lymphocyte levels act as protective factors. Previous studies (16, 19) have found that MP-infected children have more neutrophils and fewer lymphocytes compared with controls. Liu (8) observed that the neutrophil count in the blood or alveolar lavage fluid was elevated in patients with MP infection compared to those with bocavirus infection; however, neutrophils predominated in both groups. This process may be related to the role of neutrophils in immune responses, where they are involved in inflammation and strengthening host defense capabilities. Once their action is complete, neutrophils are rapidly eliminated, which reduces inflammatory responses and promotes tissue healing in pneumonia through the activity of lymphocytes and macrophages (20, 21). Accordingly, increased neutrophil levels indicate a marked inflammatory response.

In this study, we found that elevated LDH levels and fever peaks were independent risk factors for PB, and fever duration was recognized as a risk factor in univariate analysis. This finding is consistent with those reported by Zhao and Zhang (3, 4, 19). They reported higher LDH levels, longer fever durations, and higher fever peaks in patients with PB and MP pneumonia. However, PB caused by ADV infection, as reported by Yuan (22), also exhibited significantly increased LDH levels and recurrent fever. We believe that increased body temperature in children can lead to increased breathing and heart rate without a corresponding increase in water loss. When fluid intake is insufficient, mucus secretion can thicken, making the development of PB easier. In cases of pneumonia or airway obstruction in children, ischemia and hypoxia trigger anaerobic glycolysis, causing a significant release of LDH into the blood. Elevated LDH levels are strongly linked to the severity and prognosis of mycoplasma and adenovirus pneumonia, as shown by meta-analysis studies (23, 24).

In univariate analysis, the risk factors for PB included atelectasis and pleural effusion; however, multivariate analysis suggested that atelectasis was an independent risk factor, whereas pleural effusion was not. This finding is consistent with that of Zhao (19). While Zhang reported that pleural effusion is an independent risk factor for PB, he did not include atelectasis in his analysis (3). Among the 69 cases of PB reported by Lin (25), 35 had pleural effusion (50.72%), whereas 34 did not. In the pleural effusion group, C-reactive protein and LDH levels were significantly higher, suggesting a potential relationship with the degree of the inflammatory reaction (25). Our results conclude that pleural effusion and atelectasis are risk factors for PB, with atelectasis being more specifically linked to this condition. This is related to PB, characterized by infection or other factors that lead to the production of large, gelatinous, or rigid branched airway casts in the trachea. These casts can cause partial or complete airway obstruction, resulting in atelectasis.

Currently, the mechanisms underlying PB remain unclear; however, the most effective and direct treatment method is to remove the plastic foreign body obstructing the airway using bronchoscopy (12, 17). Bronchoscopy is an invasive procedure that involves risks such as mucosal hemorrhage and hypoxemia (26). Besides, it is relatively difficult to identify PB in children with pneumonia at an early stage, making it extremely important to evaluate its presence (27). In this study, a nomogram was created and confirmed to predict PB development in pediatric pneumonia cases using multivariate logistic regression to identify five key factors: MP infection, fever spike, N%max, and atelectasis. The nomogram demonstrated good performance, reflecting its accuracy and discriminatory ability. These five data points, accessible during a child's hospital stay, assist clinicians in early PB detection. Suppose the following two situations: 1. Consider a patient with Mycoplasma positivity, a peak temperature of 39.8°C, 85% neutrophils, atelectasis on CT, and an LDH level of 680 U/L. The scoring is as follows: Mycoplasma: 45 points, Temperature: 60 points, Neutrophils: 60 points, Atelectasis: 55 points, LDH: 40 points. Total score: 260 points, indicating a 0.91 probability and high risk, requiring immediate treatment. 2. For another patient who is Mycoplasma negative, with the same peak temperature of 39.8°C, 70% neutrophils, no atelectasis, and an LDH level of 680 U/L, the scoring is as follows: Mycoplasma: 0 points, Temperature: 60 points, Neutrophils: 50 points, Atelectasis: 0 points, LDH: 40 points. Total score: 150, indicating a 0.05 probability and low risk. For very low PB risk, prioritize drug therapy. For high-risk children, it is strongly recommended to perform early bronchoscopy to prevent complications.

However, our model has certain limitations. Firstly, this investigation was a retrospective analysis conducted at a single center. Despite rigorously adhering to the inclusion and exclusion criteria outlined in the study and employing multiple logistic regression to minimize confounding factors, the potential for selection bias remains. Secondly, our pathogen testing was confined to bacterial culture results from MP, RSV, ADV, and alveolar lavage fluid. Given that clinical presentations often involve mixed infections, there is a possibility of concurrent infections with other undetected viruses or bacteria, thereby imposing limitations on our findings. Lastly, the model is based on data from a single center with a relatively small sample size and lacks external validation, which may constrain its generalizability. Consequently, conducting multicenter and prospective studies is recommended as a subsequent step to enhance and refine the model.

## 5 Conclusion

A nomogram for MP infection, fever peak, LDH, N%max, and atelectasis was established to predict the risk of PB in children with pneumonia. This model will be helpful for guiding active bronchoscopy in children with pneumonia. But prospective external validation is needed before putting this model into practice.

## Data Availability

The original contributions presented in the study are included in the article/Supplementary Material, further inquiries can be directed to the corresponding author.
